# Supplementary material for: 3D-MINFLUX nanoscopy reveals distinct allosteric mechanisms for activation and modulation of PIEZO1 by Yoda1
Source: Nat Commun. 2025 Dec 17;16:11192. doi: 10.1038/s41467-025-67610-x (PMC12712055; doi:10.1038/s41467-025-67610-x)
Supplement: Supplementary file 1 — Supplementary Information [file 41467_2025_67610_MOESM1_ESM.pdf]

# Supplementary Information

**Title:** 3D-MINFLUX nanoscopy reveals distinct allosteric mechanisms for activation and modulation of PIEZO1 by Yoda1

**Authors:** Clement Verkest[1], Lucas Roettger[1], Nadja Zeitzschel[1] & Stefan G. Lechner[1,*]

**Affiliations:**

[1] Department of Anaesthesiology, University Medical Center Hamburg-Eppendorf, Germany

*Corresponding Author. Email: s.lechner@uke.de

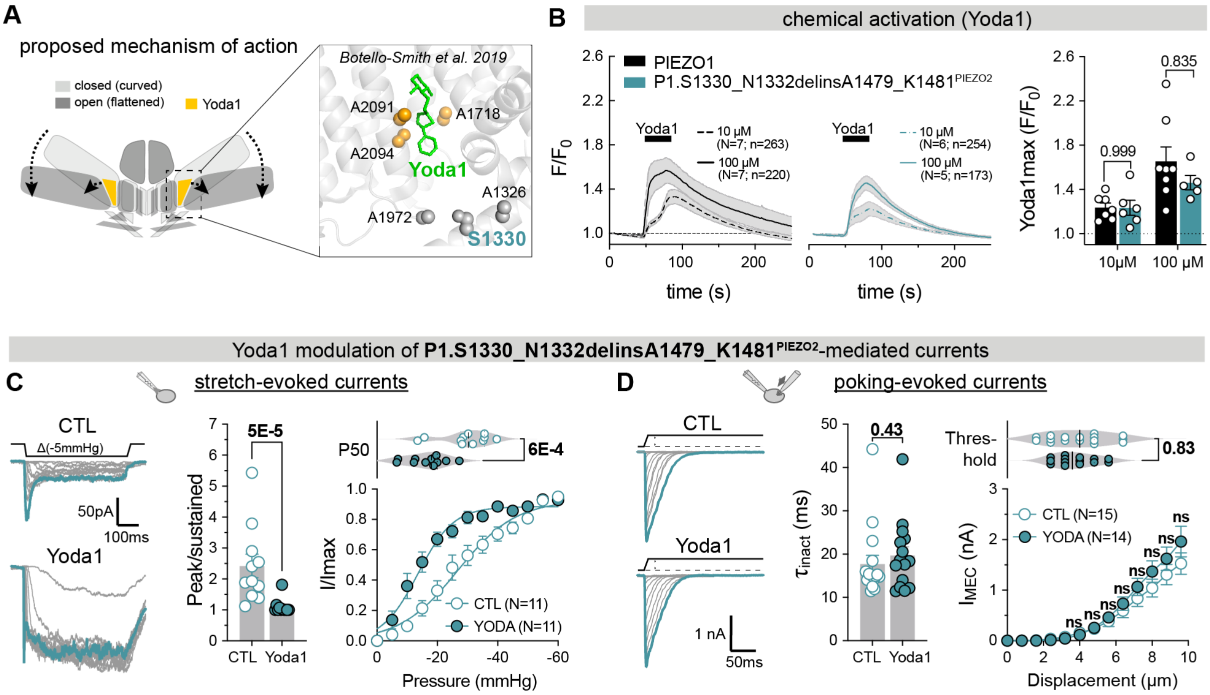


**Supplementary Figure 1 | Mutation of a possible Yoda1 binding site at the beam–THU8/9 interface does not alter Yoda1 sensitivity (A)** Cartoon depicting the proposed mechanism of action of Yoda1 on PIEZO1 (left) and close-up view of the THU8–9 interface with previously proposed residues lining putative Yoda1 binding sites highlighted in sphere representation. **(B)**, time courses of mean ± SEM $Ca^{2+}$-influx ($F/F_0$, GCamp8) evoked by 10 and 100 µM Yoda1 in cells expressing PIEZO1 (left) and P1.S1330_N1332delinsA1479_K1481$^{PIEZO2}$ (center) and comparison (two-sided Mann-Whitney test) of the mean ± SEM maximum responses (right). 'N' denotes the numbers of coverslips and 'n' denotes the total number of cells. Bars (right panel) represent means ± SEM and circles show the mean population responses of individual coverslips (N-numbers provide in bars). **(C)** Modulation of P1.S1330_N1332delinsA1479_K1481$^{PIEZO2}$ stretch-evoked currents by 30µM Yoda1. Example traces evoked by incrementing pressure stimuli (left), comparison of peak/sustained ratio (middle) using Mann-Whitney test (CTL = 2.4 ± 1.2, N=11 vs Yoda1 = 1.1 ± 0.2, N=11, P=0.00005), pressure-response curves (i.e. peak current amplitude at indicated pressure normalized to maximal response, bottom right) and comparison of $P_{50}$ values in the absence and presence of Yoda1 using Student's t-test test (CTL = -29.3 ± 8.2 mmHg, N=11 vs Yoda1 = -16.5 ± 6.5 mmHg, N=11, P=0.0006). **(D)** Modulation of P1.S1330_N1332delinsA1479_K1481$^{PIEZO2}$ poking-evoked currents in whole-cell recordings by 30µM Yoda1. Example traces evoked by incrementing (Δ 800nm, left), comparison of inactivation time constants obtained with exponential decay fit (middle) using XY test (CTL = 17.7 ± 8.2 ms, N=16 vs Yoda1 = 19.64 ± 7.9 ms, N=15, P=0.429), displacement-response curves (i.e. peak current amplitude vs. indicated stimulus magnitude; bottom, right) and comparison of mechanical activation thresholds using Mann-Whitney test (CTL = 3.95 ± 1.6 µm, N=16 vs Yoda1 = 3.77 ± 1.1, N=14, P=0.825).

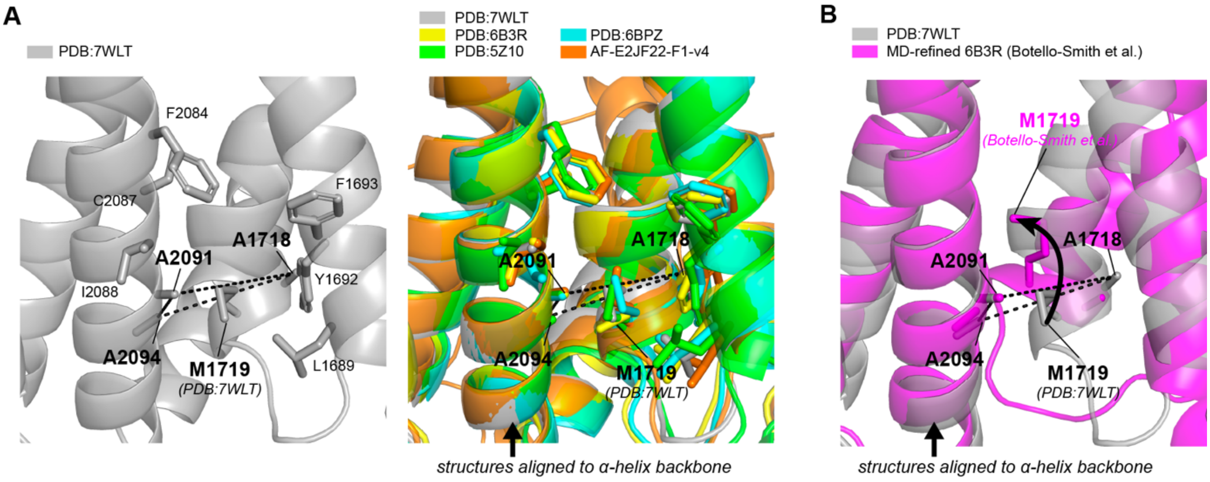


**Supplementary Figure 2 | Comparison of THU8–9 interface in available PIEZO1 structures.** **(A)** left, close-up side view of the THU8–9 interface in PDB:7WLT with residues A2091, A2094 and A1718, supposedly lining the Yoda1 curved-state binding site highlighted in stick representation. Note, the space between the three residues is occupied by the side chain of M1719. Right, overlay of all previously resolved PIEZO1 cryo-EM structures (7WLT, 6B3R, 5Z10, 6BPZ) as well as the Alphafold prediction (AF-E2JF22-F1-v4), demonstrating the same structural arrangement of side chains in all five structures. Note, for better comparison the structures were aligned to the indicated helix of the 7WLT structure. **(B)**, Overlay of the 7WLT structure used for binding pocket and docking pose analysis here (7WLT, grey) and the molecular dynamics (MD)simulation-refined structure previously analysed by Botello-Smith and colleagues. Note, there is a subtle yet relevant difference in the orientation of the M1719 side chain and the α-helix in which it resides, such that the space between A2091, A2094 and A1718 is vacant in the MD-refine structure allowing Yoda binding.

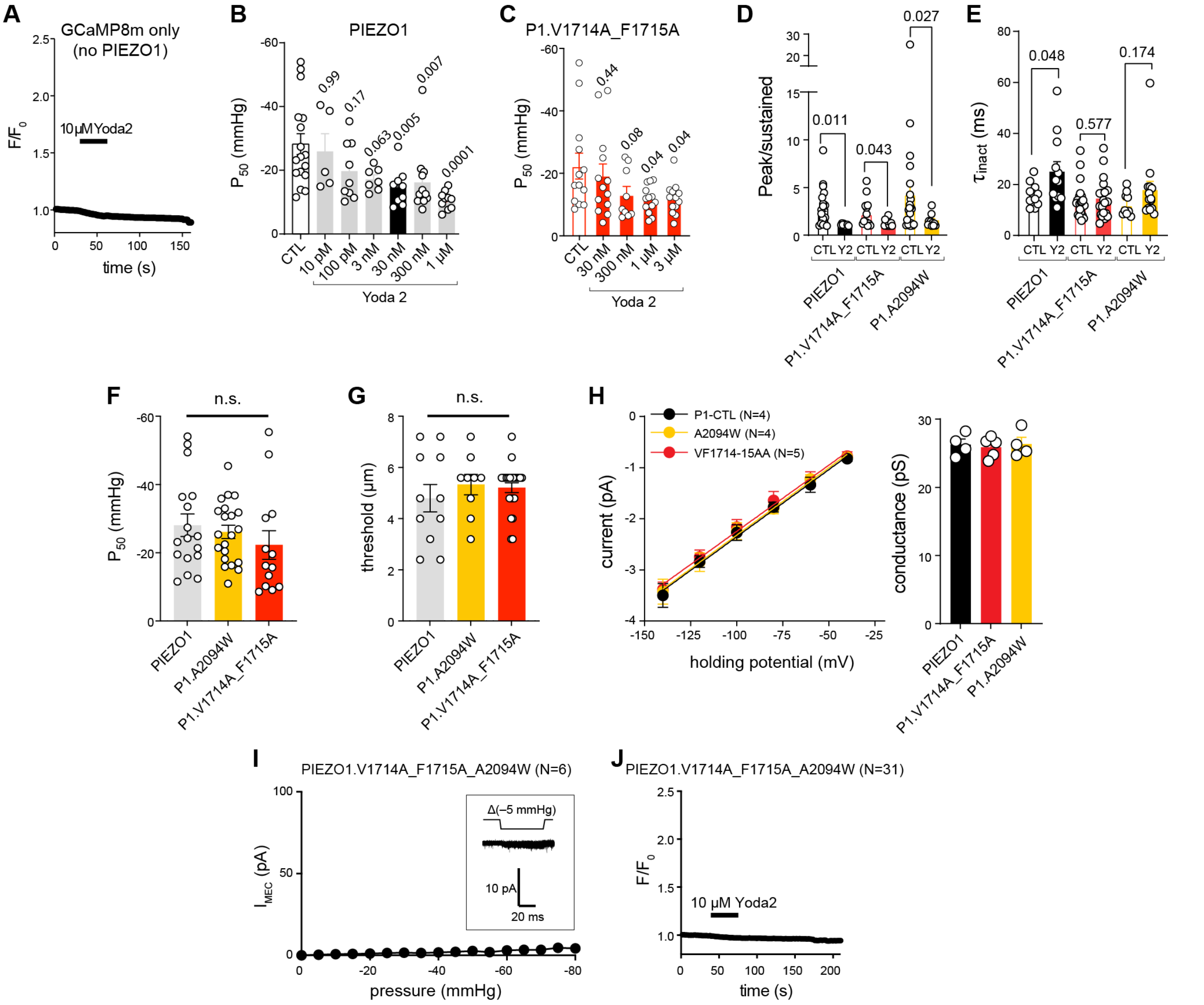


**Supplementary Figure 3 | Electrophysiological characterization of Yoda2 modulatory effect on PIEZO1 and associated mutants. (A)** time course of mean ± SEM $Ca^{2+}$-influx ($F/F_0$, GCamp8) evoked by 10 µM Yoda2 in control cells expressing only GCamp8 but no PIEZO1. Note, Yoda does not produce any calcium influx in the absence of PIEZO1 indicating that responses shown in Fig. 3 exclusively result from Yoda2-induced activation of PIEZO1 and PIEZO1 mutants, respectively. **(B)** Comparison of the mean ± s.e.m. P50 for PIEZO1 Control (N=17) and Yoda2 at various concentration (N=5-12), determined from stretch-evoked currents in N2a-P1KO cells. **(C)** Comparison of the mean ± s.e.m. P50 for PIEZO1.V1714A_F1715A Control (N=13) and Yoda2 at 300nM (N=9), Mann-Witney test CTL vs 300nM (P=0.0433). **(D)** Kinetic analysis of stretch-evoked currents. Comparison of the mean ± s.e.m. Peak/sustained ratio for PIEZO1 and associated mutants in the presence or absence of 30nM Yoda2, Mann-Witney test. **(E)** Kinetic analysis of whole-cell poking-evoked currents. Comparison of the mean ± s.e.m. inactivation time constant for PIEZO1 and associated mutants in the presence or absence of 30nM Yoda2, unpaired t-test. **(F)** Comparison of the control (untreated) P50 from stretch-evoked currents of PIEZO1, A2094W and V1714A_F1715A, one-way ANOVA (P=04429. **(G)** Comparison of the control (untreated) threshold of whole-cell poking-evoked currents of PIEZO1, A2094W and V1714A_F1715A, one-way ANOVA (P=0.5769). **(H)** Current/voltage relationship of single-channel amplitude of PIEZO1, A2094W and V1714A_F1715A, fitted with a linear regression (left). Comparison of the mean ± s.e.m. single channel conductance (right), Kruskall-Wallis test (P=0.9523). **(I)** Displacement-response curve of mechanically-evoked currents recorded from N2a-P1KO cells transfected with a PIEZO1.V1714A_F1715A_A2094W triple mutant. Inset shows example recording. Note, the PIEZO1 triple mutant is completely insensitive to mechanical stimuli. **(J)**, time course of mean ± SEM $Ca^{2+}$-influx ($F/F_0$, GCamp8) evoked by 10 µM Yoda2 in cells expressing a PIEZO1.V1714A_F1715A_A2094W triple mutant. Note, the PIEZO1 triple mutant is completely insensitive to Yoda2.

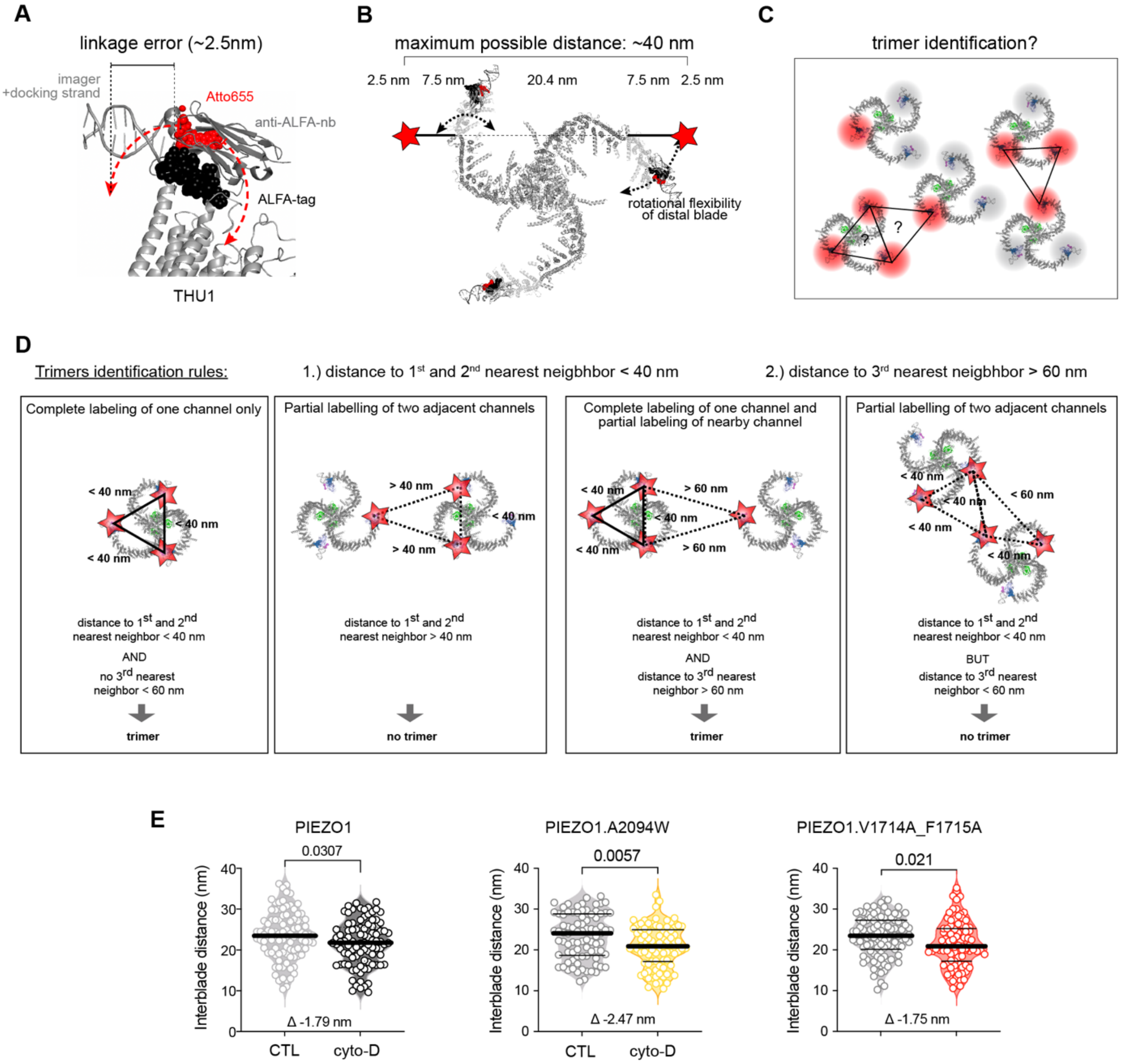


**Supplementary Figure 4 | PIEZO trimer identification with 3D-MINFLUX. (A**) Cartoon illustrating the labelling strategy and its associated linkage error. The position of the extracellular ALFA tag is depicted, with its associated nanobody with a DNA strand and the complementary DNA strand with Atto655 fluorophore. **(B)** Representation of a full-length PIEZO1 (top view) and the possible maximum interblade distance measured experimentally. **(C)** Schematic representation of the trimer identification problem due to partial labelling and proximity of neighbouring PIEZO1 channels. **(D)** Trimer identification pipeline. **(E)** Comparison of the mean ± s.e.m. interblade distance of the identified trimers in untreated cells or after addition of cytochalasin-D for PIEZO1 (N=98 and 93), A2094W (N=85 and 86) and V1714A_1715A (N=107 and 91), with two-sided Student's unpaired t-test or Mann-Witney test (A2094W).

| Primers | 5' --> 3' | Template | Generated construct |
|---|---|---|---|
| P1-A2094W-fw | atttactttgccctgtcctggtaccagatccgctgtggc | PIEZO1-mScarlet | PIEZO1-A2094W-mScarlet |
| P1-A2094W-rv | gccacagcggatctggtaccaggacagggcaaagtaaat | | |
| P1-A2094W-fw | atttactttgccctgtcctggtaccagatccgctgtggc | PIEZO1-ALFA-mGreenLantern | PIEZO1-ALFA-A2094W-mGL |
| P1-A2094W-rv | gccacagcggatctggtaccaggacagggcaaagtaaat | | |
| P1-KSIN-P2-fw | aatgcagccaacctgaaggcggtcaaGttccatcgccagattgag | PIEZO1-mScarlet | PIEZO1-S1330-N1332del-insA1479-K1481-P2 |
| P1-KSIN-P2-rv | ctcaatctggcgatggaaCttgatcgccttcaggttggctgcatt | | |
| P1-VF1714-15AA-fw | gtgctgcccgtgcttgCgGCcctgtgggccatgctg | PIEZO1-mScarlet | PIEZO1-VF1714-15AA-mScarlet |
| P1-VF1714-15AA-rv | cagcatggcccacaggGCcGcaagcacgggcagcac | | |
| P1-VF1714-15AA-fw | gtgctgcccgtgcttgCgGCcctgtgggccatgctg | PIEZO1-ALFA-mGreenLantern | PIEZO1-ALFA-VF1714-15AA-mGL |
| P1-VF1714-15AA-rv | cagcatggcccacaggGCcGcaagcacgggcagcac | | |
| P1-VF1714-15AA-fw | gtgctgcccgtgcttgCgGCcctgtgggccatgctg | PIEZO1-A2094W-mScarlet | PIEZO1-VF1714-15AA-A2094W-mScarlet |
| P1-VF1714-15AA-rv | cagcatggcccacaggGCcGcaagcacgggcagcac | | |

**Supplementary Table 1, DNA-primers used for cloning**

| Iteration | Modality | Pattern diameter (nm) | photon limit | back-ground limit | dwell time (ms) | pattern repeat | Stickiness | CFR limit | laser power factor |
|---|---|---|---|---|---|---|---|---|---|
| 0 | hexagonal | 251 | 160 | 15000 | 1 | 1 | - | none | 1 |
| 1 | zline | 251 | 400 | 15000 | 1 | 1 | 2 | none | 1 |
| 2 | square | 251 | 100 | 10000 | 1 | 5 | 2 | none | 1 |
| 3 | zline2 | 251 | 50 | 10000 | 1 | 5 | 2 | none | 1 |
| 4 | square | 132 | 67 | 10000 | 1 | 5 | 2 | 0.9 | 2 |
| 5 | zline2 | 132 | 33 | 10000 | 1 | 5 | 2 | none | 2 |
| 6 | square | 66 | 67 | 10000 | 1 | 5 | 2 | 0.8 | 4 |
| 7 | zline2 | 66 | 33 | 10000 | 1 | 5 | 2 | none | 4 |
| 8 | square | 35 | 100 | 10000 | 1 | 5 | 2 | none | 6 |
| 9 | zline2 | 35 | 50 | 10000 | 1 | 5 | 2 | none | 6 |

**Supplementary Table 2, 3D-MINFLUX scan parameters**
