# Supplementary material for: 3D-MINFLUX nanoscopy reveals distinct allosteric mechanisms for activation and modulation of PIEZO1 by Yoda1
Source: Nat Commun. 2025 Dec 17;16:11192. doi: 10.1038/s41467-025-67610-x (PMC12712055; doi:10.1038/s41467-025-67610-x)
Supplement: Supplementary file 2 — Reporting Summary [file 41467_2025_67610_MOESM2_ESM.pdf]

nature portfolio

Corresponding author(s): Stefan Lechner

Last updated by author(s): Nov 25, 2025

Reporting Summary

Nature Portfolio wishes to improve the reproducibility of the work that we publish. This form provides structure for consistency and transparency in reporting. For further information on Nature Portfolio policies, see our Editorial Policies and the Editorial Policy Checklist.

Statistics

For all statistical analyses, confirm that the following items are present in the figure legend, table legend, main text, or Methods section.

n/a	Confirmed	
☐	☒	The exact sample size (*n*) for each experimental group/condition, given as a discrete number and unit of measurement
☐	☒	A statement on whether measurements were taken from distinct samples or whether the same sample was measured repeatedly
☐	☒	The statistical test(s) used AND whether they are one- or two-sided<br>*Only common tests should be described solely by name; describe more complex techniques in the Methods section.*
☒	☐	A description of all covariates tested
☐	☒	A description of any assumptions or corrections, such as tests of normality and adjustment for multiple comparisons
☐	☒	A full description of the statistical parameters including central tendency (e.g. means) or other basic estimates (e.g. regression coefficient) AND variation (e.g. standard deviation) or associated estimates of uncertainty (e.g. confidence intervals)
☐	☒	For null hypothesis testing, the test statistic (e.g. *F*, *t*, *r*) with confidence intervals, effect sizes, degrees of freedom and *P* value noted<br>*Give P values as exact values whenever suitable.*
☒	☐	For Bayesian analysis, information on the choice of priors and Markov chain Monte Carlo settings
☒	☐	For hierarchical and complex designs, identification of the appropriate level for tests and full reporting of outcomes
☒	☐	Estimates of effect sizes (e.g. Cohen's *d*, Pearson's *r*), indicating how they were calculated

Our web collection on statistics for biologists contains articles on many of the points above.

Software and code

Policy information about availability of computer code

Data collection	Electrophysiology: Patchmaster (V2x91, HEKA)<br>MINFLUX: abberior imspector v16.3
Data analysis	The following sotfwares were used for data analysis and/or collection:<br>Matlab v2024a (Mathworks)<br>SwissDock AutoDock Vina (www.swissdock.ch)<br>Binding pocket analysis (https://proteins.plus/)<br>FitMaster (V2x91, HEKA)<br>IgorPro 8 (WaveMetrics)<br>Excel (V16.16.27; Microsoft)<br>Prism Graphpad 8 (GraphPad Software)<br>Fiji/ImageJ (v2.3.0/1.53f; NIH)<br>PyMol 2.4.0 (Schrödinger, LLC)

For manuscripts utilizing custom algorithms or software that are central to the research but not yet described in published literature, software must be made available to editors and reviewers. We strongly encourage code deposition in a community repository (e.g. GitHub). See the Nature Portfolio guidelines for submitting code & software for further information.

Data

Policy information about availability of data

All manuscripts must include a data availability statement. This statement should provide the following information, where applicable:

- Accession codes, unique identifiers, or web links for publicly available datasets
- A description of any restrictions on data availability
- For clinical datasets or third party data, please ensure that the statement adheres to our policy

All data supporting the article, such as the MINFLUX and patch-clamp analysis outputs for each experimental condition, are provided as source data. Raw data and reagents that are not commercially available are available from the corresponding author upon reasonable request. Source data are provided with this paper.

Research involving human participants, their data, or biological material

Policy information about studies with human participants or human data. See also policy information about sex, gender (identity/presentation), and sexual orientation and race, ethnicity and racism.

Reporting on sex and gender	n/a
Reporting on race, ethnicity, or other socially relevant groupings	n/a
Population characteristics	n/a
Recruitment	n/a
Ethics oversight	n/a

Note that full information on the approval of the study protocol must also be provided in the manuscript.

Field-specific reporting

Please select the one below that is the best fit for your research. If you are not sure, read the appropriate sections before making your selection.

☒ Life sciences ☐ Behavioural & social sciences ☐ Ecological, evolutionary & environmental sciences

For a reference copy of the document with all sections, see nature.com/documents/nr-reporting-summary-flat.pdf

Life sciences study design

All studies must disclose on these points even when the disclosure is negative.

Sample size	sample size was not predetermined. Sample size was chosen based on our experience and experiments from published literature
Data exclusions	no data was excluded from the analysis
Replication	All experiments in this study were performed independently with at least five independent biological replicates (i.e. cells), yielding similar results.
Randomization	Randomization is inherent to MINFLUX imaging and patch-clamp recordings, because all cells in the culture look alike and hence the experimenter inevitably chooses cells without any bias and absolutely random from the pool of healthy, well-attached and fluorescent (transfected) cells.
Blinding	For logistic reasons the experimenter who performed the N2a cell transfections also had to perform the MINFLUX and the patch-clamp recordings. Hence, blinding was not possible.

Reporting for specific materials, systems and methods

We require information from authors about some types of materials, experimental systems and methods used in many studies. Here, indicate whether each material, system or method listed is relevant to your study. If you are not sure if a list item applies to your research, read the appropriate section before selecting a response.

Materials & experimental systems

n/a	Involved in the study
☐	☒ Antibodies
☐	☒ Eukaryotic cell lines
☒	☐ Palaeontology and archaeology
☒	☐ Animals and other organisms
☒	☐ Clinical data
☒	☐ Dual use research of concern
☒	☐ Plants

Methods

n/a	Involved in the study
☒	☐ ChIP-seq
☒	☐ Flow cytometry
☒	☐ MRI-based neuroimaging

Antibodies

Antibodies used	Massive-Tag-Q Anti-ALFA, Massive-Tag-Q Anti-GFP (Massive Photonics).
Validation	The Massive-Tag nanobodies were validiated in a previous study (https://doi.org/10.1101/2024.11.26.625366)

Eukaryotic cell lines

Policy information about cell lines and Sex and Gender in Research

Cell line source(s)	Piezo1 knock-out mouse Neuro2a cells were a gift from Dr. Gary R Lewin (produced from N2a, ATCC CCL-131 from ATCC;Reference: PMID: 29545531)
Authentication	None of the cell lines used were authenticated.
Mycoplasma contamination	Cell line was not tested for mycoplasma contamination
Commonly misidentified lines (See ICLAC register)	No commonly misidentified cell line was used in this study.

Plants

Seed stocks	n/a - bug in form!!! field does not disappear when checking the corresponding box above.
Novel plant genotypes	n/a
Authentication	n/a
